# Regeneration of the digestive system in the crinoid *Himerometra robustipinna* occurs by transdifferentiation of neurosecretory-like cells

**DOI:** 10.1371/journal.pone.0182001

**Published:** 2017-07-28

**Authors:** Nadezhda V. Kalacheva, Marina G. Eliseikina, Lidia T. Frolova, Igor Yu. Dolmatov

**Affiliations:** 1 A.V. Zhirmunsky Institute of Marine Biology, National Scientific Center of Marine Biology, Far Eastern Branch, Russian Academy of Sciences, Vladivostok, Russia; 2 Far Eastern Federal University, Vladivostok, Russia; Laboratoire de Biologie du Développement de Villefranche-sur-Mer, FRANCE

## Abstract

The structure and regeneration of the digestive system in the crinoid *Himerometra robustipinna* (Carpenter, 1881) were studied. The gut comprises a spiral tube forming radial lateral processes, which gives it a five-lobed shape. The digestive tube consists of three segments: esophagus, intestine, and rectum. The epithelia of these segments have different cell compositions. Regeneration of the gut after autotomy of the visceral mass progresses very rapidly. Within 6 h after autotomy, an aggregation consisting of amoebocytes, coelomic epithelial cells and juxtaligamental cells (neurosecretory neurons) forms on the inner surface of the skeletal calyx. At 12 h post-autotomy, transdifferentiation of the juxtaligamental cells starts. At 24 h post-autotomy these cells undergo a mesenchymal-epithelial-like transition, resulting in the formation of the luminal epithelium of the gut. Specialization of the intestinal epithelial cells begins on day 2 post-autotomy. At this stage animals acquire the mouth and anal opening. On day 4 post-autotomy the height of both the enterocytes and the visceral mass gradually increases. Proliferation does not play any noticeable role in gut regeneration. The immersion of animals in a 10^−7^ M solution of colchicine neither stopped formation of the lost structures nor caused accumulation of mitoses in tissues. Weakly EdU-labeled nuclei were observed in the gut only on day 2 post-autotomy and were not detected at later regeneration stages. Single mitotically dividing cells were recorded during the same period. It is concluded that juxtaligamental cells play a major role in gut regeneration in *H*. *robustipinna*. The main mechanisms of morphogenesis are cell migration and transdifferentiation.

## Introduction

The study of the mechanisms of cell fate determination and cell reprogramming is one of the basic trends in modern biology [1]. This knowledge is important for progress in transplantology, regenerative medicine and cancer research. An example of the most radical cell reprogramming is transdifferentiation. Eguchi and Kodama [2] defined transdifferentiation (direct reprogramming) as a functional switching of cells from one differentiated state to another. Most publications refer to studies of mammalian cells, in which transdifferentiation most frequently occurs in the case of pathological processes or carcinogenesis [3–9]. Natural transdifferentiation is found in many species of invertebrates and lower vertebrates at sexual or asexual reproduction, as well as during regeneration [1,10–16]. Therefore, these organisms provide convenient models for the study of transdifferentiation mechanisms.

Echinoderms possess extensive regenerative capacity. They can regenerate both small appendages and large regions of the body after substantial damage such as division of the body into two or three parts [17–24]. There have been many attempts to explain these abilities by the involvement of stem cells in echinoderms [25,26]. Nevertheless, no reliable evidence for the presence of stem cells in these animals, apart from primordial germ cells, has been provided [27]. A possible exception is the cells that give rise to coelomocytes [28]. Any direct evidence for participation of stem cells in regeneration in echinoderms is also absent. On the other hand, numerous data show that in these animals lost organs are formed from differentiated cells in the remaining tissues [18,19,21,22,29–32]. In addition, it was convincingly demonstrated that, after the complete removal of the gut in the holothurian *Eupentacta fraudatrix* (D'yakonov & Baranova in D'yakonov et al., 1958), the lost organ develops due to the transdifferentiation of coelomic epithelial cells [13].

Crinoids are the most ancient of extant echinoderms. These animals can regrow arms, cirri, pinnules, internal organs, as well as the entire viscera [17,33–38]. Currently, the most detailed information is available on the regeneration of arms in crinoids after autotomy or other injury [17,34]. Recovery occurs by the process of epimorphosis, i.e. through the regrowth of the remaining parts of organs. Amoeboid cells that are normally arranged around the radial nerve and, apparently, migrate to the damaged area are considered to be stem cells [25].

Regeneration of the gut in crinoids is of particular interest as regards the study of the cellular sources involved in regeneration. In these animals, the complex of internal organs, which is referred to as the visceral mass, is located in the cup-shaped skeletal calyx and can easily be removed. As a result, crinoids lose the entire digestive system, i.e. all the structures of endodermal origin. Nevertheless, these animals restore lost organs after such serious damage quite rapidly. This phenomenon has been mostly neglected. Over the last 130 years, only four articles have been published describing the regeneration of the visceral mass in crinoids [37,39–41]. Of these publications, only the last provides a detailed cytological analysis of the mechanisms of gut formation. According to it, no involvement of undifferentiated cells was found in regeneration after the artificial removal of the visceral mass in *Antedon mediterranea* (Lamarck, 1816). It was suggested that the digestive epithelium in this species is formed from coelomic epithelium cells as a result of their transdifferentiation.

Recently it was shown that the comatulid *Himerometra robustipinna* (Carpenter, 1881) possesses the ability to autotomize its visceral mass [42]. This involves the rupture of the connective tissue layer that separates the sub-intestinal and aboral coeloms. It was also demonstrated that juxtaligamental cells (JLCs) are involved in this process. JLCs are granule-containing effector cells that control the mechanical properties of echinoderm collagenous tissues [43,44]. When autotomy occurs, all organs of endodermal origin are completely removed, and only the mesenteries of the aboral coelom remain on the inner surface of the skeletal calyx. In spite of this, the lost structures are completely restored within 7 days [45]. In this regard, *H*. *robustipinna* is an interesting model for investigating mechanisms of transdifferentiation. The present work describes regeneration of the digestive system after autotomy in this species. Furthermore, since the structure of the normal gut in *H*. *robustipinna* has not been studied to date, special attention was paid to the structure and cellular morphology of the digestive tube.

## Material and methods

### Animals

Adult red feather stars, *Himerometra robustipinna* (Carpenter, 1881) (Crinoidea, Comatulida), were collected in Nha Trang Bay, South China Sea, from a depth of 3–5 m. Then the animals were kept in a tank with running aerated seawater. The water temperature during the period of experiments was 27–29°C. *H*. *robustipinna* are abundant in coastal areas of Vietnam. The species is not endangered or protected. They are invertebrate animals and no specific permissions are required for their collection. Autotomy of the visceral mass was provoked as described previously [42]. Six visceral masses were used to study the normal structure of the digestive system immediately after autotomy. Calyces with regenerating visceral mass were fixed at various times after autotomy. Before fixation, cirri and most of the arms were removed.

### Light microscopy

The material for light microscopy was fixed in 4% formaldehyde solution in seawater. The animals were stored in this solution for 1–2 months at 4°C prior to processing. The material was decalcified with 5% EDTA solution in 4% formaldehyde for 14 days, then washed in water for 1 h, and dehydrated in a series of increasing concentrations of ethanol and in chloroform. Then the specimens were embedded in paraffin. Sections 5–6 μm thick were cut on a microTec CUT 4050 microtome and stained with hematoxylin and eosin.

### 3D reconstruction

Paraffin sections were used for reconstruction of the shape of the digestive system. For 3D reconstruction, every section was photographed to generate a stack of images. Alignment, tracing of contours of interest, and 3D surface representations were achieved using Amira software.

### Electron microscopy

The material was fixed for 2–3 h in 2.5% glutaraldehyde solution in 0.05 M cacodylate buffer (pH 7.4) at a temperature of 4°C. To study the gut structure, the autotomized visceral masses were immersed entirely in the fixative. Prior to fixation, a few drops of the fixative were applied to the calyx of the regenerating animals for immobilization and pre-fixation of tissues. Then arms and cirri were removed, and the calyx was immersed entirely in the fixative. To study ultrastructural features of gut regeneration, the animals were fixed at 6, 12, and 18 h, and on days 1, 2, 4, and 7 post-autotomy. Three samples were used at each regenerative stage. The material was stored in the same fixative for 1–2 months at 4°C prior to processing. Subsequently, the material was washed in 0.05 M cacodylate buffer (pH 7.4) at 4°C and post-fixed for 1 h with 1% OsO_4_ solution prepared in the same buffer. For scanning electron microscopy samples were dehydrated through increasing concentrations of ethanol and acetone and dried under CO_2_. The dried specimens were mounted on SEM pin stubs, coated with platinum and analyzed using a Sigma 300 VP scanning electron microscope.

For transmission electron microscopy the material was decalcified for 25 days in several changes of a solution containing 1% ascorbic acid and 0.15M NaCl [46]. The material was dehydrated in increasing concentrations of ethanol, then acetone, and embedded in a mixture of Araldite M and Epon 812 (Fluka) according to the standard technique [47]. Sections were made using a Reichert Ultracut E ultramicrotome. Semithin 0.7 μm sections were stained with 1% methylene blue in a 1% aqueous solution of sodium tetraborate. Their analysis and photography were performed using a Jenamed 2 (Carl Zeiss Jena) light microscope, equipped with a Nikon D1x digital camera. Ultrathin 60 nm sections were stained with 1% uranyl acetate in 10% ethanol, then with lead citrate, and analyzed using Libra 120 and Libra 200FE (Carl Zeiss) transmission electron microscopes.

### Treatment with colchicine

For the experiments we used a solution of the mitotic inhibitor colchicine in seawater at a concentration of 10^-3^–10^-7^M. After autotomy and on days 4 and 7 after the removal of the visceral mass, animals were placed into 2-liter aquariums with the colchicine solution and kept there for 2 days. The water was aerated throughout the experiment. For each period, three individuals were selected. The control animals were kept in aquariums of the same volume filled with aerated seawater without colchicine. The material was fixed and stored in 2.5% glutaraldehyde solution in 0.05 M cacodylate buffer (pH 7.4) at 4°C. The further processing of specimens and cutting of semithin and ultrathin sections were performed as described above.

### Identification of DNA-synthesizing cells

Cells in the S period of the mitotic cycle were detected using a Click-iT EdU kit (Invitrogen, Molecular Probes). The visceral mass was removed from 9 animals, which then were kept in tanks with aerated running seawater. On days 2, 4, and 7 post-autotomy, 3 specimens (on each of the days) were placed in a 10 μM EdU solution in sterile seawater for 2 h. The calyces of the animals with anlages (primordia) of the visceral mass were fixed in 4% paraformaldehyde in PBS (pH 7.5) for 5 h at 4°C; then the material was washed in PBS and stored in the same buffer at 4°C. Before processing, the anlage of the visceral mass was cut off from the calyx with a scalpel, soaked in a 15% sucrose solution in PBS, and placed in the medium NEG-50 (Thermo Scientific, USA) for making frozen sections. Sections 14–16 μm in thickness were cut on a HM 560 Cryo-Star freezing microtome (Thermo Scientific, USA). The reaction for EdU detection was performed according to the manufacturer’s protocol.

### Immunocytochemistry

After detection of EdU, sections were incubated in a solution of monoclonal antibodies against alpha-tubulin (SIGMA, USA) in 3% BSA in PBS (1:1000) at 40°C for 12 h. The sections were then washed in three portions of PBS and post-stained for 1 h with secondary antibodies against mouse immunoglobulins labeled with Alexa 546 (Invitrogen, Molecular Probes), diluted 1:1500 on 1% BSA in PBS. Subsequently, the material was washed in three portions of PBS and embedded in anti-fade Vestfaled medium (Invitrogen, Molecular Probes), containing DAPI for detection of DNA. The material was analyzed using a LSM 780 (Carl Zeiss) confocal laser scanning microscope. Green fluorescence (488 nm) indicated EdU inclusion in the DNA of the cells; red fluorescence marked alpha-tubulin, localized predominantly in cilia; blue fluorescence, nuclear DNA.

Material treatment and analyses were performed in the “Far Eastern Center of Electron Microscopy” (National Scientific Center of Marine Biology, Far Eastern Branch, Russian Academy of Sciences, Vladivostok, Russia)

## Results

### Structure of the digestive system

**Fig 1:**
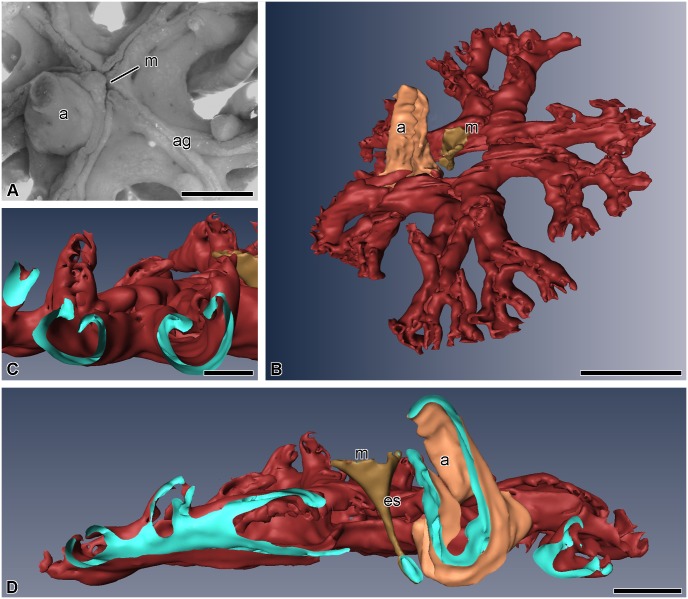
Three-dimensional reconstruction of the digestive system of *H*. *robustipinna*. (A) General view of the visceral mass (light microscopy). (B) View from oral side. (C) Transverse section (oral-aboral) of radial lateral outgrowth of the gut. (D) Transverse section (oral-aboral) of the gut through central part. a, anal cone; ag, ambulacral groove; es, esophagus; m, mouth; the pale blue color indicates the internal surfaces of the visceral mass in sites of cut. Scale bar: (A and B) 5 mm, (C) 2 mm, (D) 1 mm.

In *H*. *robustipinna*, the mouth orifice is located in the center of the visceral mass (Fig 1A and 1B). It has the shape of a narrow arcuate slit, one end of which is reached by the ambulacral grooves of the radii B and C, and the other end by D and E. The groove of the radius A adjoins the convex side of the mouth slit. The anal cone is located at the concave side, in the CD interradius, on the periphery of the calyx. The digestive system consists of three segments: esophagus, intestine, and rectum. The mouth orifice is followed by the esophagus, which descends from the center of the calyx down to its aboral side and passes into the intestine (Fig 1D). The latter forms one coil clockwise, then rises to the oral side, and ends as the rectum located in the anal cone. The intestine forms radial lateral outgrowths, reaching the bases of arms, which impart the five-lobed shape to the visceral mass (Fig 1B). The gut opens as the anal orifice at the top of the anal cone. All along its length, the intestinal tube is flattened in the oral-aboral direction. Its lateral parts are curved and raised up, which imparts a horseshoe shape to the gut in a cross section (Fig 1C).

**Fig 2:**
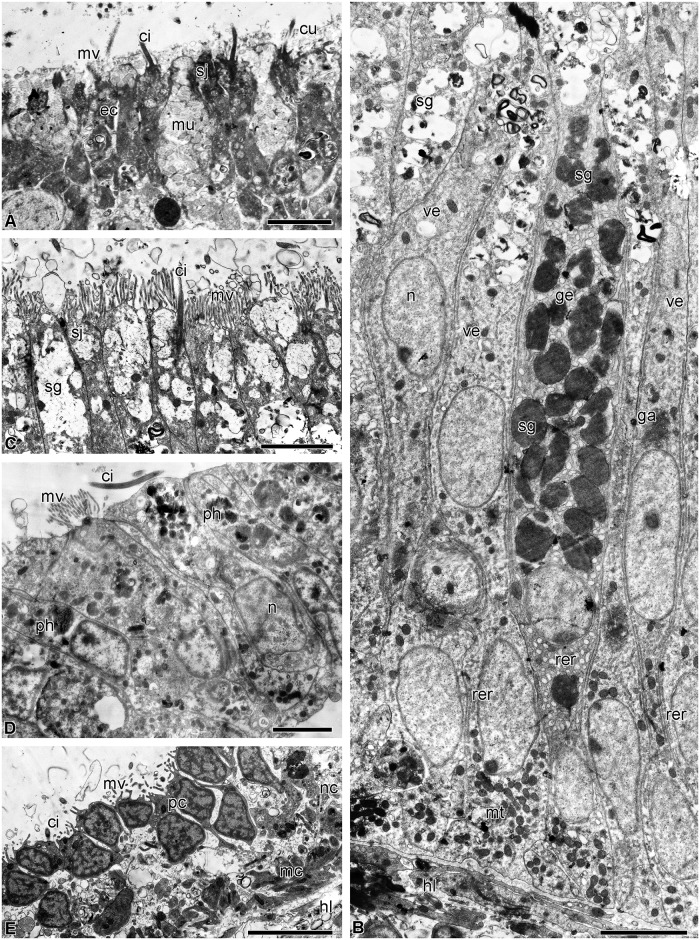
Structure of the luminal epithelium of the digestive tube of *H*. *robustipinna*. (A) Esophagus. (B) General view of the intestinal epithelium. (C) Apical part of the intestinal epithelium. (D) The epithelium of the rectum. (E) Coelomic epithelium of the gut. ci, cilium; cu, cuticle; ec, epitheliocyte; ga, Golgi apparatus; ge, granular enterocyte; hl, hemal lacuna; mu, mucus cell; mc, myoepithelial cell; mt, mitochondria; mv, microvilli; n, nucleus; nc, processes of nerve cells; pc, peritoneocyte; ph, phagosome; rer, rough endoplasmic reticulum; sj, septate junction; sg, secretory granules; ve, vesicular enterocyte. Scale bars: (A) 2 μm, (B-E) 4 μm.

The esophagus is lined with a cuticular epithelium consisting of ciliated epitheliocytes and mucus cells (Fig 2A). The height of the epithelium gradually increases from 10 to 40 μm as the distance from the mouth increases. The subcuticular space is filled with a substance of average electron density, which is probably mucus. Ciliated epitheliocytes are narrow cells. In the apical portion, they are connected via desmosomes and septate junctions. The septate junctions can reach a length of 1.5 μm. The cell contains a large nucleus with a nucleolus; large clumps of heterochromatin are well discernible in karyoplasm. In the cytoplasm there are oval mitochondria, a small Golgi apparatus (GA) situated near the nucleus, cisternae of rough endoplasmic reticulum (RER) and free ribosomes. The apical surface bears numerous microvilli.

The mucus cells of the esophagus have a wide apical portion filled with granules containing a heterogeneous substance of medium electron density (Fig 2A). The nucleus is located in the basal portion of the cell. The apical surface bears a cilium and numerous microvilli.

The intestinal epithelium has a height of 40 μm and forms numerous folds all along its length. It consists of tall, narrow cells of two types (Fig 2B). The most numerous are known as vesicular enterocytes [48]. Their apical portion is filled with large secretory granules containing a substance of low electron density with a reticulate structure. The cells are connected with one another via desmosomes and septate junctions. On the apical surface there are numerous microvilli and each cell bears a cilium (Fig 2C). The nuclei are rounded in shape and contain small clumps of heterochromatin. The well developed GA is located in the perinuclear region (Fig 2B). In the cytoplasm, there are narrow RER cisternae and free ribosomes. A large number of mitochondria are found in the basal portion of cells (Fig 2B).

The second type of intestinal epithelial cells are known as granular enterocytes [48]. On their apical surface they also have numerous microvilli and a cilium. These cells are filled with large electron-dense granules surrounded by a membrane (Fig 2B). In addition, granular enterocytes have a large number of rounded RER cisternae and free ribosomes in the cytoplasm. The nucleus contains a nucleolus and small clumps of heterochromatin (Fig 2B).

The epithelium of the rectum is formed by cells 20 μm in height (Fig 2D). They contain an irregularly shaped nucleus with large clumps of heterochromatin, a small GA, RER and free ribosomes (Fig 2D). The surface of some cells bears microvilli and cilia. The enterocytes of the rectum do not contain secretory granules, but phagosomes are found in their cytoplasm (Fig 2D). In the area of the anal orifice, the luminal epithelium of the gut merges with the integumentary epidermis.

The bodies and processes of nerve cells are distributed in the basal part of the luminal epithelium all along the intestine. Under the basal lamina of the epithelium of the digestive system there are hemal lacunae. The coelomic epithelium of the gut is represented by peritoneocytes and myoepithelial cells with bundles of axons of the basiepithelial nerve plexus located between them (Fig 2E).

**Fig 3:**
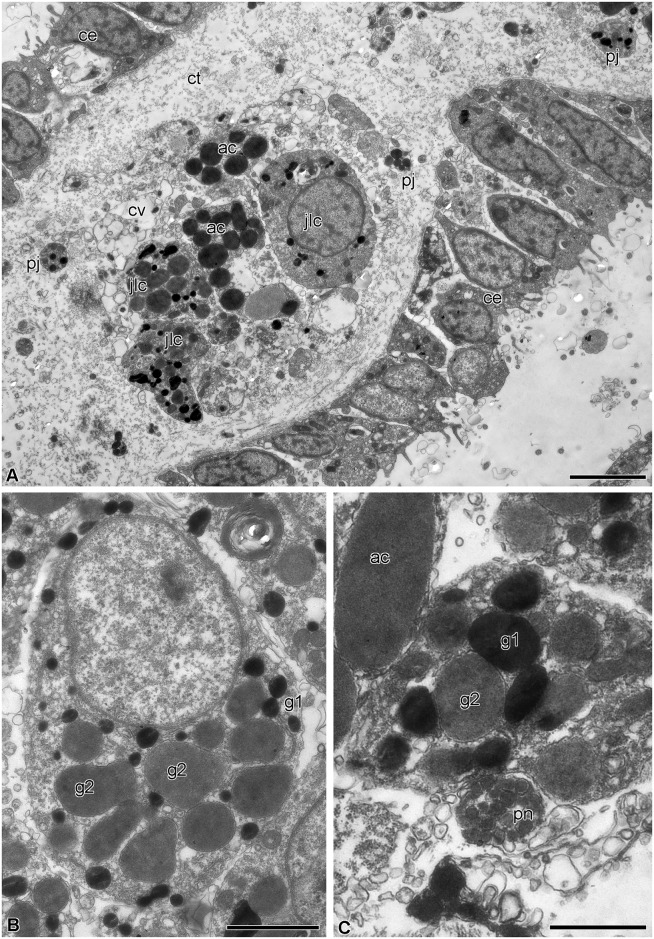
Structure of the septa of the aboral coelom of *H*. *robustipinna*. (A) General view of septa. (B) Juxtaligamental cell. (C) Close apposition of process of juxtaligamental cell and axon (pn). ac, amoebocyte; ce, coelomic epithelium; ct, connective tissue; cv, cell with electron-transparent vacuoles; g1, type 1 granule; g2, type 2 granule; jlc, luxtaligamental cell; pj, process of juxtaligamental cell. Scale bars: (A) 4 μm, (B) 2 μm, (C) 1 μm.

The visceral mass is attached to the calyx by septa of the aboral coelom. Although the structure of the septa has already been described in detail [42], a short overview is provided here because of the critical involvement of certain septal components in gut regeneration. The septa are thin connective tissue partitions covered by coelomic epithelium (Fig 3A); their oral region is attached to the aboral wall of the subintestinal coelom. Various cell types are present in the septal connective tissue, including juxtaligamental cells (JLCs) containing two types of cytoplasmic granules: type 1 has contents of high electron density and is roughly circular in profile with a diameter of 0.2–0.5 μm; type 2 has a circular to oval profile, contents of medium electron density and size (0.6–1 μm) × (0.3–0.8 μm) (Fig 3B). Nerve cells and axons are also present in the septal connective tissue, some axons being close to the processes and bodies of JLCs (Fig 3C).

### Regeneration of the digestive system

**Fig 4:**
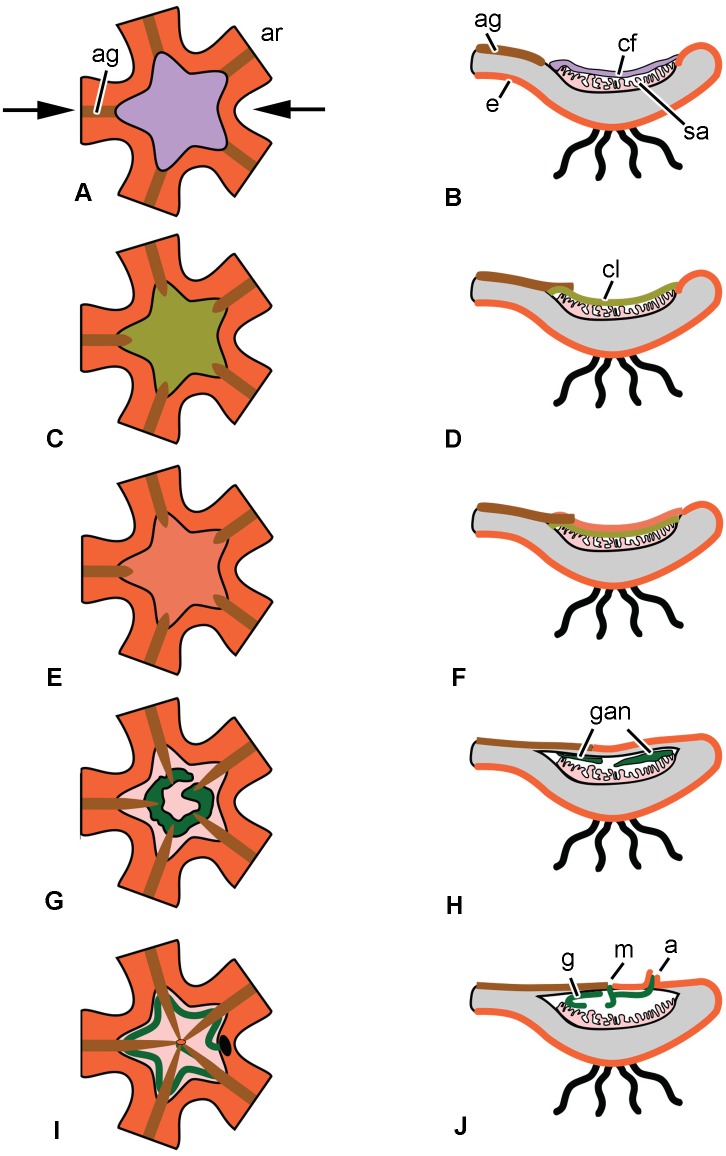
Scheme of the consecutive stages of the regeneration of the digestive system in *H*. *robustipinna*. (A, C, E, G, I)–oral view, (B, D, F, H, J)–transverse section of the calyx (arrows in (A) indicate location of the section plane, (G, I)–epidermis on oral side is not shown). (A, B) Immediately after autotomy. (C, D) 6–12 h post-autotomy. (E, F) 18–24 h post-autotomy. (G, H) 2 days post-autotomy. (I, J) 4 days post-autotomy. a, anal cone; ag, ambulacral groove; ar, arm; cf, coagulated coelomic fluid; cl, cellular layer; e, epidermis; g, gut; gan, gut anlage; m, mouth; sa, septa of the aboral coelom.

A characteristic feature of the regeneration of the visceral mass in the comatulid *H*. *robustipinna* is the rapidity of the process. As early as day 4 after the complete removal of viscera, the animal acquires a mouth orifice and anal cone. This means that by this time the digestive system is largely reformed and the animal is able to feed. The successive stages of regeneration of the digestive system in *H*. *robustipinna* are depicted diagrammatically in Fig 4.

#### 6–12 h post-autotomy

**Fig 5:**
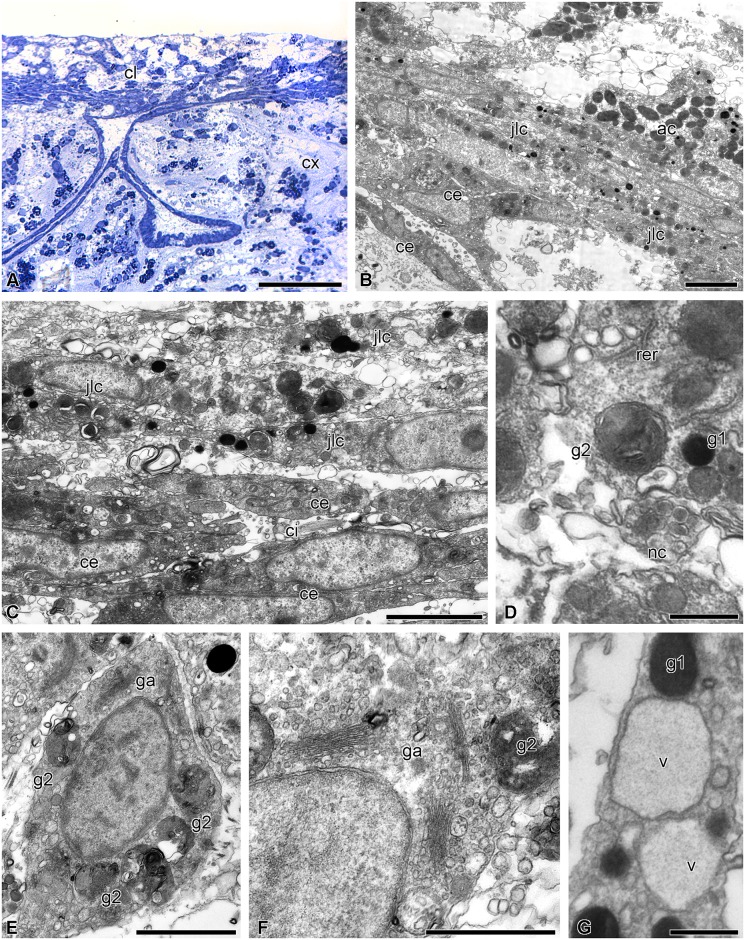
Microscopic organization of the cellular layer covering the surface of the calyx at 6–12 h post-autotomy in *H*. *robustipinna*. (A) Transverse (oral-aboral) semithin section of the oral part of the calyx at 6 h post-autotomy. (B) Cellular layer on the surface of the calyx at 6 h post-autotomy. (C) Juxtaligamental cells covering the torn septa of the aboral coelom at 6 h post-autotomy. (D) Transforming type 2 granules at 12 h post-autotomy. (E) Juxtaligamental cell with type 2 granules undergoing destruction at 12 h post-autotomy. (F) Golgi apparatus in the cytoplasm of juxtaligamental cell at 12 h post-autotomy. (G) Large vacuoles in the cytoplasm of juxtaligamental cell at 12 h post-autotomy. ac, amoebocyte; ce, coelomic epithelial cell; ci, cilium; cl, cellular layer; cx, calyx; g1, type 1 granule; g2, type 2 granule; ga, Golgi apparatus; jlc, juxtaligamental cell; nc, process of nerve cell; rer, rough endoplasmic reticulum; v, vacuole. Scale bars: (A) 50 μm, (B) 4 μm, (C, E, F) 2 μm, (D, G) 0.5 μm.

This period is characterized by the process of wound closure and formation of a cellular layer on the oral surface of the calyx from which the visceral mass will subsequently develop. Immediately after autotomy, the calyx surface is covered by a layer of coagulated coelomic fluid, which is likely to protect the damaged structures from infection by microorganisms (Fig 4A and 4B) [42]. The process of migration of cells of various types apparently begins during the first hours after autotomy, because the loose cellular layer, having a thickness of 40–50 μm and covering the torn septa of the aboral coelom on the surface of the calyx, is formed within 6 h. It consists of JLCs, amoebocytes, and coelomic epithelial cells (Figs 4C, 4D and 5A–5C).

At 12 h post-autotomy, the cellular layer becomes denser. Its thickness is now about 60 μm. Of all the cell types in the layer, changes in JLCs are most noticeable. In terms of morphology these changes appear as the destruction of type 2 granules. The granules are surrounded by a double membrane and transformed into autophagosomes (Fig 5D and 5E). The contents of the granules acquire a heterogeneous organization. Along with the destruction of type 2 granules, the synthetic apparatus of the JLCs also appears to become more active. The number of RER cisternae and free ribosomes in their cytoplasm increases; a well-developed GA is found in the perinuclear region of the cells (Fig 5E and 5F). RER cisternae exhibit synthesis and accumulation of a substance of low electron density. As a result, large irregularly shaped vacuoles containing this substance appear in the cytoplasm of JLCs (Fig 5G). Type 1 granules remain unchanged at this stage (Fig 5G).

#### 18–24 h post-autotomy

**Fig 6:**
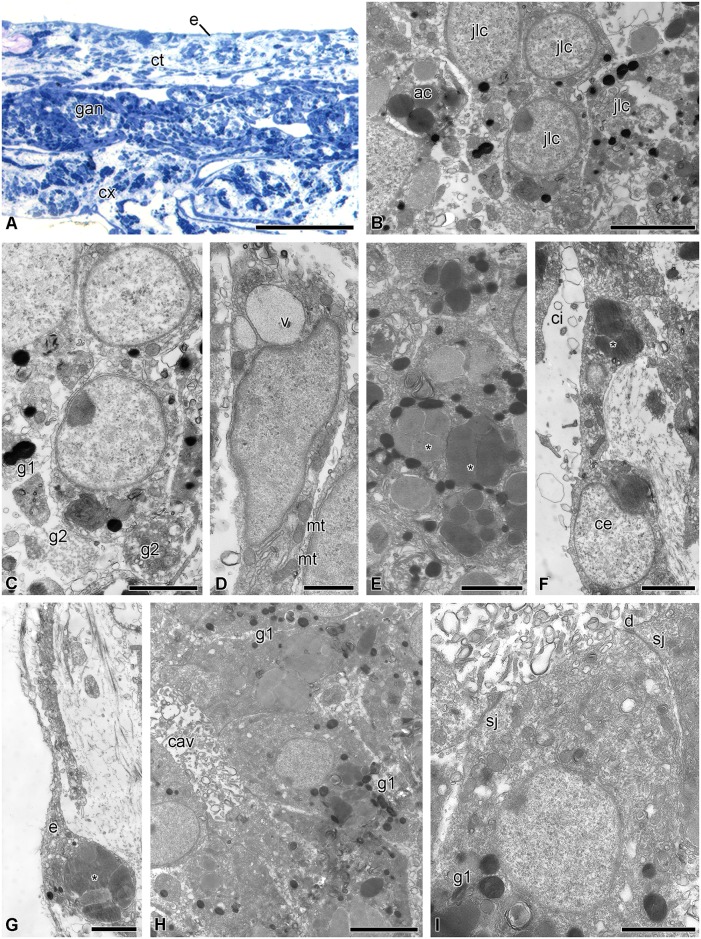
Gut anlage at 18–24 h post-autotomy in *H*. *robustipinna*. (A) Transverse (oral-aboral) semithin section of the cellular layer on the calyx surface at 18 h post-autotomy. (B) Aggregation of juxtaligamental cells and amoebocytes in the gut anlage at 18 h post-autotomy. (C) Juxtaligamental cell with type 2 granules undergoing destruction at 18 h post-autotomy. (D) Polarizing juxtaligamental cell at 18 h post-autotomy. (E) Juxtaligamental cells with amoebocyte granules (*). (F) Cell of coelomic epithelium with amoebocyte granules (*). (G) Epidermal cell with amoebocyte granules (*). (H) Intestinal epithelium composed of transformed juxtaligamental cells at 24 hours post-autotomy. (I) Transformed juxtaligamental cell at 24 h post-autotomy. ac, amoebocyte; cav, cavity; ce, coelomic epithelial cell; ci, cilium; ct, connective tissue; cx, calyx; d, desmosome; e, epidermis; g1, type 1 granule; g2, type 2 granule; gan, gut anlage; jlc, juxtaligamental cell; mt, mitochondrion; sj, septate junction; v, vacuole. Scale bars: (A) 50 μm, (B, H) 4 μm, (C, E-G, I) 2 μm, (D) 1 μm.

This stage is characterized by the beginning of gut formation. At 18 h post-autotomy, the cellular layer on the calyx surface is divided into two parts (Figs 4E, 4F and 6A). The external part is the future tegmen (the outermost dorsal layer of the visceral mass). It consists of epidermis and a connective tissue layer. The epidermis is represented by flattened epidermal cells that migrated here probably from the periphery of the calyx. Their apical surface bears short microvilli. The extracellular matrix is composed of sparse collagen fibers and an amorphous component. In addition, amoebocytes and JLCs having a uniform structure are found in the connective tissue. The extracellular matrix is separated from the inner part of the aggregation by the coelomic epithelium of the forming subintestinal coelom.

The internal portion of the cellular aggregation is the gut anlage. The aboral side of the anlage is separated from the surrounding structures by the coelomic epithelium of the subintestinal coelom. The inner portion of the anlage is filled by aggregations of JLCs and amoebocytes (Fig 6B). JLCs continue to change, although this transformation is not synchronous in all cells. Thus, in some JLCs destruction of type 2 granules still continues (Fig 6C), whereas these granules are no longer present in the cytoplasm of other cells (Fig 6D). Cells at a later stage of transformation show signs of polarization: in that part of them that faces the inner region of the aggregation, large vacuoles containing a substance of low electron density accumulate (Fig 6D). A large number of mitochondria are observed in the opposite part of the cells.

The destruction of amoebocytes located in the forming visceral mass begins at 18 h post-autotomy. The granules of the amoebocytes are now located in the extracellular matrix where they are phagocytosed by surrounding cells. As a result, starting from this moment and during subsequent regeneration events, epidermal cells, cells of the coelomic epithelium and transforming JLCs all contain phagosomes enclosing amoebocyte granules (Fig 6E–6G).

At 24 h post-autotomy, the aggregations of JLCs increase in size. There are cavities in the internal parts of the aggregations (Fig 6H). This causes formation of the intestinal epithelium to begin. Transformed JLCs (which are precursors of enterocytes) that comprise it are connected with one another via desmosomes and septate junctions (Fig 6I). The apical surface of the cells bears solitary microvilli. Type 2 granules disappear; only the residual bodies of autophagosomes can be seen in the cytoplasm (Fig 6I). In addition, the large vacuoles containing a substance of low electron density are now absent from cells. However, at this stage of regeneration, type 1 granules are still retained in their cytoplasm (Fig 6H and 6I).

#### 2 days post-autotomy

**Fig 7:**
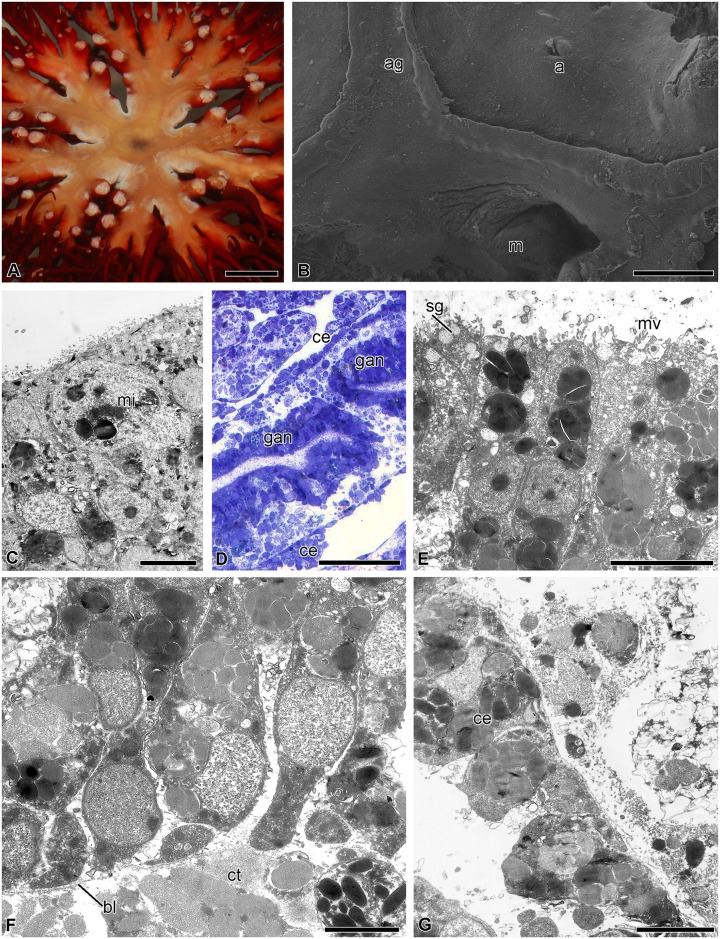
Gut anlage at 2 days post-autotomy in *H*. *robustipinna*. (A) General view of the calyx with regenerating visceral mass. (B) Mouth and anal openings. (C) Epidermis with mitotically dividing cell (mi). (D) Gut anlage. (E) Apical part of the enterocytes. (F) Basal part of the enterocytes. (G) Coelomic epithelium of the gut. a, anal opening; ag, ambulacral groove; bl, basal lamina; ce, coelomic epithelial cell; ct, connective tissue; gan, gut anlage; m, mouth; mi, mitosis; mv, microvilli; sg, secretory granules. Scale bars: (A) 5 mm, (B) 400 μm, (D) 50 μm, (C) 5 μm, (E, G) 4 μm, (F) 2 μm.

On day 2 post-autotomy, the ambulacral grooves reach the middle of the calyx (Figs 4G, 4H and 7A). They form a ring around the central portion of the calyx. The mouth is formed in the peripheral interradial part of the ring (Fig 7B). There is a small anal opening with diameter about 50 μm in the interradius near the mouth (Fig 7B). Mitotically dividing cells can sometimes be observed in the epidermis (Fig 7C). Beneath the tegmen, formation of the digestive system still continues. The size of the aggregations of enterocyte precursors increases as a result of their merging together (Fig 7D). The cells composing them, connected via desmosomes and septate junctions, become arranged in a certain order to form the true epithelium. On the apical surface of the cells, the number of microvilli increases (Fig 7E). The epithelium is separated from the surrounding connective tissue by a well-defined basal lamina (Fig 7F). Secretory granules characteristic of mature enterocytes appear in the apical portion of the cytoplasm. Type 1 granules are no longer detected in the cytoplasm of the enterocyte precursors. Beneath the intestinal epithelium there is a layer of extracellular matrix. A large number of disintegrating granules of amoebocytes are observed in it (Fig 7F). The lining of the subintestinal coelom consists of peritoneocytes, the cytoplasm of which is filled with amoebocyte granules (Fig 7G).

#### 4 days post-autotomy

**Fig 8:**
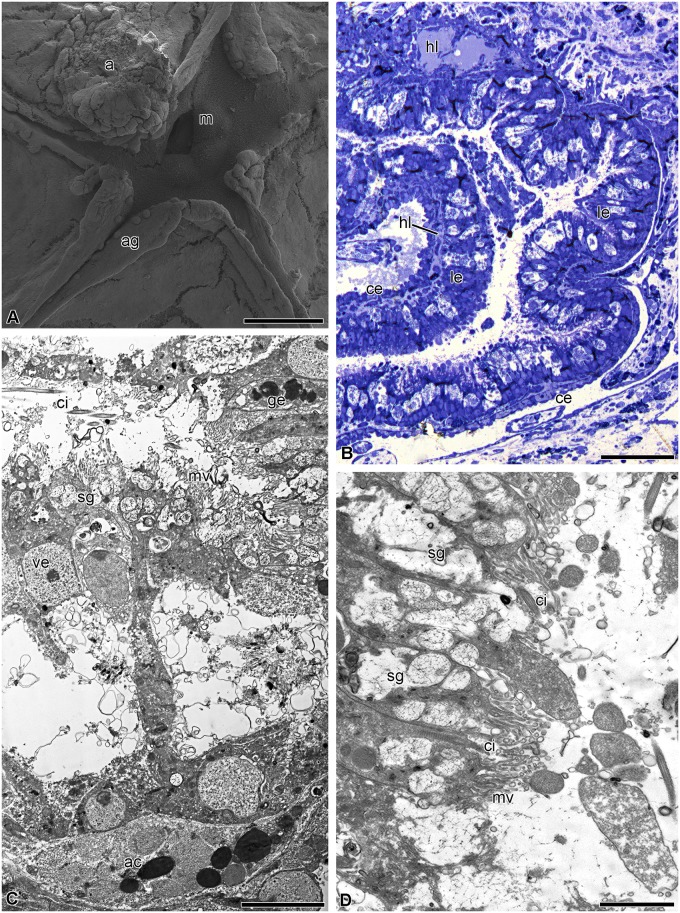
Digestive system at 4 days post-autotomy in *H*. *robustipinna*. (A) General view of oral surface of the visceral mass. (B) Digestive tube. (C) Luminal epithelium. (D) Apical part of the enterocytes. a, anal cone; ac, amoebocyte; ag, ambulacral groove; ce, coelomic epithelium; ci, cilium; ge, granular enterocyte; hl, hemal lacuna; le, luminal epithelium; m, mouth; mv, microvilli; sg, secretory granule; ve, vesicular enterocyte. Scale bars: (A) 400 μm, (B) 20 μm, (C, D) 4 μm.

On day 4 post-autotomy, the ambulacral grooves reach the center of the calyx and become connected to the mouth (Figs 4I, 4J and 8A). A small anal cone appears in one of the interradii. The luminal epithelium of the gut forms folds; its height is about 10–15 μm (Fig 8B). It consists of vesicular enterocytes, among which single granular enterocytes occur (Fig 8C). In the luminal epithelium of the gut, there are numerous cavities that have probably formed due to the death of precursor cells (Fig 8B and 8C). The apical surface of the cell bears numerous microvilli; each cell has a cilium (Fig 8D). Mitochondria are located in the basal portion of the cytoplasm of the enterocytes (Fig 8C and 8D). Hemal lacunae appear between the coelomic epithelium and intestinal lining.

#### 7 days post-autotomy

**Fig 9:**
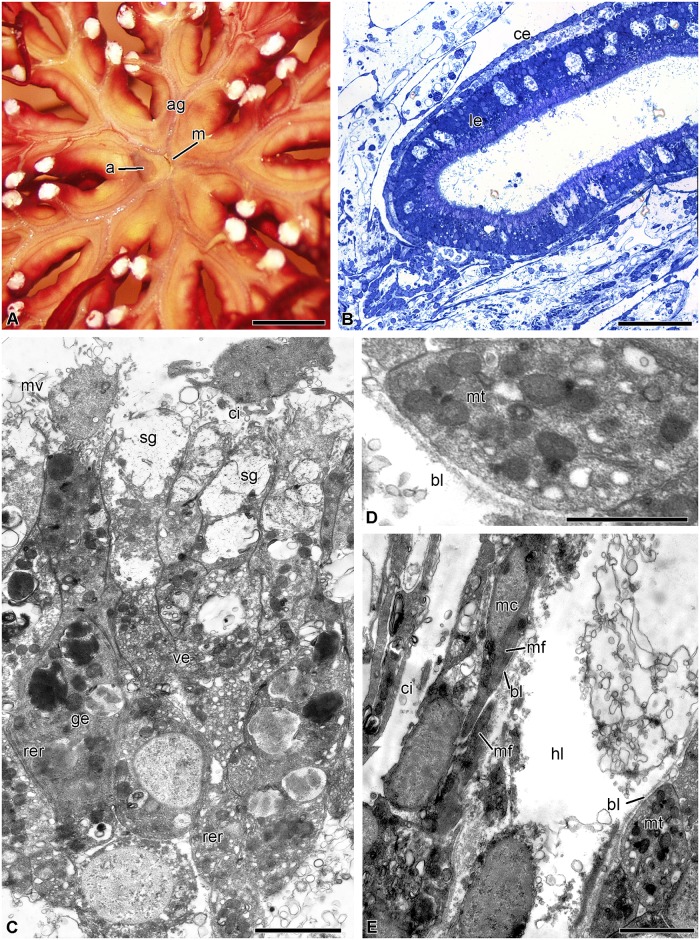
Digestive system at 7 days post-autotomy in *H*. *robustipinna*. (A) General view of the oral surface of the visceral mass. (B) Digestive tube. (C) Luminal epithelium. (D) Basal part of the enterocytes. (E) Coelomic epithelium of the gut. a, anal cone; ag, ambulacral groove; bl, basal lamina; ce, coelomic epithelium; ci, cilium; ge, granular enterocyte; hl, hemal lacuna; le, luminal epithelium; m, mouth; mc, myoepithelial cell; mf, myofilaments; mt, mitochondria; mv, microvilli; rer, rough endoplasmic reticulum; sg, secretory granule; ve, vesicular enterocyte. Scale bars (A) 5 mm, (B) 50 μm, (C) 4 μm, (E) 2 μm, (D) 1 μm.

By day 7 post-autotomy, the visceral mass grows upwards and now occupies the entire surface of the calyx. The mouth slit and the anal cone are clearly visible on its oral side (Fig 9a). The luminal epithelium of the gut at this stage of regeneration consists of long narrow enterocytes. The height of the epithelial cells reaches 40 μm, which corresponds to normal height (Fig 9b). The intestinal lining is composed of vesicular and granular enterocytes. The apical cytoplasm of the vesicular enterocytes contains numerous secretory granules (Fig 9c). A large number of mitochondria are observed in the basal portion of the cells (Fig 9d). Granular enterocytes have a typical structure (Fig 9c). Their cytoplasm contains secretory granules consisting of an electron-dense substance. Both the number of cavities and their sizes in the intestinal lining are smaller. Myoepithelial cells appear in the coelomic epithelium. Their long processes contain bundles of myofilaments (Fig 9e).

#### Cell proliferation

**Fig 10:**
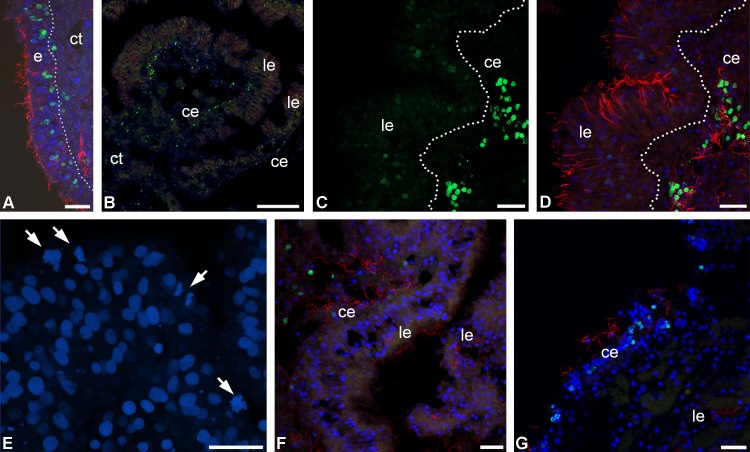
DNA-synthesizing and mitotic cells in the regenerating visceral mass of *H*. *robustipinna*. (A) Epidermis at 2 days post-autotomy; dotted line indicates basal lamina. (B) Transverse section (oral-aboral) of the radial lobe of the visceral mass at 2 days post-autotomy. (C, D) Luminal and coelomic epithelia of the gut at 2 days post-autotomy; C, EdU-labeled nuclei (green channel); D, merged channels. (E) Luminal epithelium with mitoses (arrows) at 2 days post-autotomy. (F) Luminal and coelomic epithelia of the gut at 4 days post-autotomy. (G) Luminal and coelomic epithelia of the gut at 7 days post-autotomy. ce, coelomic epithelium; ct, connective tissue; e, epidermis; le, luminal epithelium; green color, EdU; red color, tubuline; blue color, DAPI. Scale bars: (A, C-E) 20 μm, (B) 100 μm.

On day 2 post-autotomy, nuclei that included EdU were revealed in the anlage of the visceral mass of the examined animals. Intensively labeled nuclei were located in the coelomic epithelium of the aboral and subintestinal coeloms and in the epidermis (Fig 10A–10D). In the luminal epithelium of the gut, single weakly labeled nuclei were found (Fig 10C). There were also mitotically dividing cells here (Fig 10E). At the same time, no mitoses were observed in the coelomic epithelium and epidermis.

On day 4 post-autotomy, EdU-labeled nuclei were detected only in the coelomic epithelium and epidermis of the experimental animals (Fig 10F). Labeled cells were absent from the luminal epithelium of the gut. Also, no mitotically dividing cells were found in the sections. The same distribution of DNA-synthesizing cells was noted for crinoids on day 7 post-autotomy (Fig 10G). As at the previous regeneration stage, neither DNA-synthesizing cells nor mitoses were recorded from the luminal epithelium of the gut.

**Fig 11:**
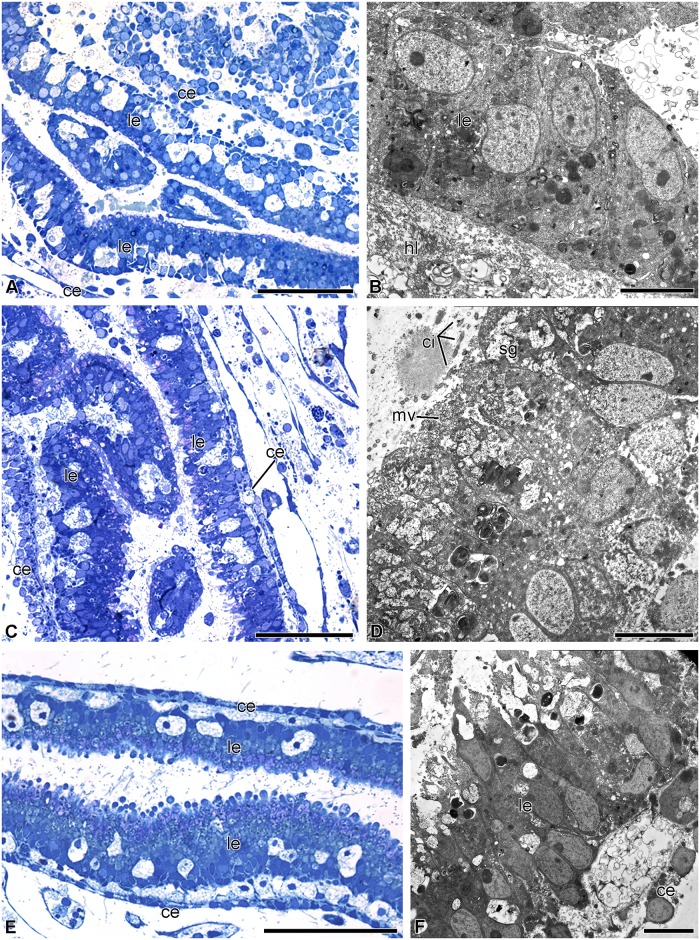
Structure of gut of *H*. *robustipinna* regenerating 2 days in 10^−7^ M colchicine solution. (A, B) The gut of the animals placed in the solution immediately after autotomy. (C, D) The gut of the animals placed in the solution on day 4 post-autotomy. (E, F) The gut of the animals placed in the solution on day 7 post-autotomy. General view of the gut (A, C, E) and fine structure of the luminal epithelium (B, D, F). ce, coelomic epithelium; ci, cilium; hl, hemal lacuna; le, luminal epithelium; mv, microvilli; sg, secretory granules. Scale bars: (A, C, E) 50 μm, (B, D, F) 4 μm.

Colchicine at a concentration of 10^−6^ M and higher caused mortality of animals within 24 h. Keeping regenerating crinoids in seawater containing 10^−7^ M colchicine did not have any noticeable effect on their viability. In this case, the animals exhibited successful formation of the visceral mass. In the crinoids placed in the solution immediately after autotomy, the intestinal epithelium, which differed little from that of the control animals, formed within two days of the experiment (Fig 11A and 11B). Nevertheless, electron micrographs showed that exposure to colchicine slightly inhibited the differentiation of the enterocytes. Their apical surface lacked microvilli; secretory granules were absent from their cytoplasm. In semithin sections mitoses were not found in the luminal epithelium of the gut nor in the coelomic epithelium.

In the animals placed in a 10^−7^ M colchicine solution on day 4 post-autotomy, regeneration of the visceral mass continued. The clearly visible visceral mass with the mouth orifice located in the center was formed by day 2 after the beginning of the experiment (day 6 post-autotomy). The small but clearly discernible anal cone developed in one of the interradii. The luminal epithelium became well formed within 6–7 days post-autotomy and its structure corresponded largely to that of the luminal epithelium of normal animals (Fig 11C and 11D). Nevertheless, granular enterocytes were absent from the intestinal lining. In semithin sections, mitoses were not found. In the crinoids placed in the colchicine solution on day 7 post-autotomy, no deviations in the development of the visceral mass and also no mitotically dividing cells were observed by the end of the experiment (Fig 11E and 11F).

## Discussion

### Structural features of the digestive system in *Himerometra robustipinna*

Our study has shown that the digestive system of *H*. *robustipinna* is of the endocyclic type typical for crinoids [48,49]. The slit-shaped mouth is located in the center of the visceral mass. From the mouth the intestinal tube descends to the aboral side and forms one coil clockwise, ending as the anal cone in the CD interradius. The visceral mass has a five-lobed shape due to the outgrowths of the digestive system, which reach the bases of arms. This anatomical feature is widely distributed in some families of comatulids, particularly in the Himerometridae and Mariametridae (Dolmatov, unpublished), but it has not been described to date. We have shown for the first time that the radial outgrowths of the visceral mass comprise the gut and thus are a part of the digestive system. This branched shape probably increases both the volume of the gut and the intensity of digestion, as achieved by the diverticula and plicae in other crinoid species [48,50].

The digestive system in *H*. *robustipinna*, like that in other crinoids, is divided into three segments: esophagus, intestine and rectum [37,48]. The luminal epithelium of the esophagus is composed of ciliated epitheliocytes and mucus cells covered by cuticle. The cuticle in this case serves as a protection against possible mechanical damage and is evidence of the ectodermal origin of this segment [48,51].

The intestinal epithelium consists of two types of enterocytes, which is typical of crinoids [48]. The most numerous cells are vesicular enterocytes. Their distinguishing feature is the presence of a large number of secretory granules containing a substance of medium electron density. These granules are assumed to contain mucus consisting of sulfated proteoglycans [48]. The presence of numerous microvilli on vesicular enterocytes indicates that these cells perform extracellular digestion and absorption of nutrients. This type of enterocyte constitutes the basis of the digestive epithelium not only in crinoids ([37,48,52], present article), but also in other echinoderms [30,51,53–55]. The wide distribution of this type of cells and the fact that they are the first cells to be differentiated during development probably indicates that they are a primary cell type in the digestive epithelium of echinoderms. The granular enterocytes are morphologically similar to such cells in other echinoderms. According to Féral and Massin [51], the granules of these cells contain digestive enzymes.

The epithelium of the rectum in *H*. *robustipinna*, like that in other crinoid species [48], is represented by epitheliocytes. The presence of microvilli and a large number of phagosomes is evidence that resorption processes and intracellular digestion take place here. This is consistent with data obtained from *Oligometra serripinna* (Carpenter, 1881) [52]. The presence of cuticle in the terminal portion of the rectum may indicate the ectodermal origin of this segment of the digestive tube [48].

Hemal lacunae occur in the connective tissue of the gut wall, which is common for echinoderms [48,51]. The major portion of the coelomic epithelium of the gut in *H*. *robustipinna*, like that in other echinoderms, is composed of peritoneal, myoepithelial, and nerve cells [21,48]. The processes of myoepithelial cells in all Echinodermata form visceral muscles [21]. In *H*. *robustipinna*, they compose the muscular system of the digestive tract, which makes peristalsis of the gut possible. These muscles are best developed in the esophagus, which is correlated with its function, viz. the forward propulsion of food. Myoepithelial cells are less developed in the gut, where movement of food is likely to be performed mainly by the beating of enterocyte cilia.

### Mechanisms of regeneration of the digestive system in *Himerometra robustipinna*

#### Stages of regeneration

Our study has shown that one of the noteworthy features of the regeneration of the viscera in *H*. *robustipinna* is the rapid development of the lost structures. As early as days 4–7 post-autotomy, all organs of the digestive system become formed and functional. On the surface of the visceral mass there are ambulacral grooves, mouth slit and anal cone. The lining of the gut is composed of functioning cells, among which granular and vesicular enterocytes are observed. However, the visceral mass of the animals on day 7 post-autotomy differs from the normal one: it is less pigmented, and the lateral outgrowths are thinner. This probably explains the discrepancy between our data and those obtained by Meyer [41] as regards the time of regeneration in *H*. *robustipinna*. That author did not study the histological structure of the regenerating visceral mass in this species, and, based solely on the external features and weight parameters of the visceral mass, stated that the recovery takes 17 days. The duration of regeneration in *Antedon bifida* (Pennant, 1777) can apparently be considered “overestimated” for the same reason. According to Dendy [39], the visceral mass in this species is re-grown within 21 days. Apparently, *H*. *robustipinna* and *A*. *bifida* initially form a smaller size of visceral mass containing a functioning intestine. Subsequently, it grows gradually to recover its normal dimensions and weight. This strategy is typical for a number of other echinoderms. During the regeneration of their internal organs, holothurians and brittle stars initially form smaller but fully functional structures that gradually increase in size during further growth [20,30,56–59]. In the case of the loss of the digestive system, this allows animals to start feeding much sooner.

The entire process of regeneration of the digestive system in *H*. *robustipinna* can be divided into three main stages. **The first stage**, 0–12 h post-autotomy, is characterized by closure of the wound surface and migration of cells onto the calyx surface. This stage is often referred to as regressive due to the inflammation of damaged organs and the active removal of destroyed structures and pathogenic organisms from the wound site that take place during this time [60]. Insulation of the remaining internal organs from the external environment should be regarded as one of the most important events of this period. A major role at this stage is played by the thrombus which is formed by coagulation of body cavity fluids (coelomic, hemal, etc.) and sticking together of wandering cells or blood cells. In particular, the comatulid *A*. *mediterranea* acquires a syncytium composed of cells that have migrated to the surface of the calyx within 24 h after removal of the visceral mass [37].

In *H*. *robustipinna* a cell-based thrombus is apparently not formed. Immediately after autotomy a layer of coagulated coelomic fluid appears on the surface of the calyx [42]; within the first 6 h it is replaced by a loose aggregation of amoebocytes and JLCs. These cells may perform a protective function by isolating the calyx surface from the external environment. Subsequently, this aggregation becomes the site of formation of the visceral mass. This feature is presumably one of the mechanisms that accelerate regeneration. Another mechanism is the early start of transformation of JLCs, which is registered within 12 h post autotomy.

**The second stage** (18–24 h post-autotomy) is the beginning of the restoration process. The cellular aggregation is divided into two layers. The external layer is the future tegmen. Its epidermis still consists of dedifferentiated, flattened cells, but the connective tissue has already begun forming underneath. The inner layer is the gut anlage, in which the luminal epithelium and the lining of the gut are formed.

**The third stage** (2–4 days post-autotomy) is characterized by active morphogenesis. All the structures of the digestive system—mouth, intestinal epithelium, and anal cone with anal orifice—are formed during this period. The fully functional digestive epithelium forms in the gut anlage. Microvilli develop on the apical surface of the enterocytes and secretory granules appear in their cytoplasm. The intestinal epithelium becomes separated from the surrounding connective tissue by a well defined basal lamina. The ambulacral grooves reach the center of the calyx and connect with the mouth orifice. The animals apparently acquire the ability to feed by the end of day 4 post-autotomy, as the cells of the intestinal epithelium have already become differentiated. Granular and vesicular enterocytes can be identified among them.

Subsequently, the visceral mass continues to grow and increase its linear dimensions. Similar stages of regeneration, but with different time parameters, were identified in the study of visceral mass regeneration in *A*. *mediterranea* [37].

#### Cellular sources of gut regeneration

In the case of autotomy of the visceral mass in *H*. *robustipinna*, the gut is completely removed, and the animal loses all tissues of endodermal origin [42]. Only torn mesenteries of the aboral coelom remain on the surface of the calyx. Thus, digestive epithelium must be formed from either stem cells or differentiated cells of mesodermal or ectodermal origin. Currently, there is no direct evidence that crinoids possess stem cells [27]. They have not been found in the studies of the ultrastructure of the internal organs ([48], present article). However, according to Candia-Carnevali et al. [25], *A*. *mediterranea* has aggregations of amoeboid cells arranged around the brachial nerve cords. These authors believe that these are stem cells involved in the regeneration of the arms in this species. *H*. *robustipinna* also has similar cells around the brachial nerve cords (our unpublished data). However, these cells, as well as other non-differentiated cells, were absent from the developing visceral mass.

The active participation of JLCs in regeneration surprised us. This study paid particular attention to the structure of these cells and their transformation during the regeneration process. Until now it was believed that JLCs are a specific type of neurosecretory-like cells characteristic of echinoderms, the function of which is to alter the mechanical properties of connective tissue [43,44,61–66]. A characteristic feature of JLCs is the presence in their cytoplasm of electron-dense granules whose largest dimension varies between species but is within the range 0.1–0.85 μm. In addition, bodies and processes of JLCs are closely associated with nerve cells.

The JLCs of *H*. *robustipinna* have a structure similar to that of JLCs in other echinoderms and are located in the connective tissue of the tegmen and septa of the aboral coelom [42], as well as in the ligaments of the arms (our unpublished data). They contain rounded or oval, very electron-dense granules with a size of 0.2–0.5 μm and their processes are often closely associated with axons. Unlike individual JLCs of most other studied echinoderm species, those of *H*. *robustipinna* contain a second type of granule, which has a size of about 1 μm and is filled with a substance of medium electron density. It is interesting that individual JLCs in some species of a starfish were also found to contain two granule types, one being around 0.1 μm in size and the other around 1 μm [67]. It is likely that the two types of granules of *H*. *robustipinna* perform different functions. The very electron-dense granules (type 1) change after autotomy and are probably involved in altering the mechanical properties of the connective tissue [42]. However, they persist for a prolonged period of time in the cell cytoplasm during regeneration. The latter circumstance made it possible to trace the transformation of JLCs at the early stages of restoration of the digestive system. Type 2 granules remain intact at autotomy, but are modified and gradually disintegrate during regeneration.

JLCs are among the first cells to migrate onto the surface of the calyx. The signs of transformation in these cells are recorded as early as at 12 h post-autotomy. Type 2 granules are subject to autophagy, due to which they become completely destroyed within 24 h post-autotomy. The process of destruction of these granules is the first morphological sign of transformation (transdifferentiation) of JLCs. These granules may contain factors that trigger the reprogramming of the JLC genome. In the process of transformation, the number of RER cisternae and free ribosomes in the cytoplasm of the JLCs increases, indicating increased synthetic activity in the cells. At 24 h post-autotomy, these cells can be considered to be precursors of the enterocytes. They gather into groups, and form intercellular junctions. The cells become polarized: microvilli develop on their surface facing the internal region of the aggregation (future apical surface). At the opposite, basal portion, the number of mitochondria increases. Subsequently, on days 2–4 post-autotomy, a typical intestinal epithelium, consisting of vesicular and granular enterocytes, is formed from them.

Besides JLCs, other cell types, particularly coelomic epithelial cells and amoebocytes, are also identified in the anlage of the visceral mass. We have not investigated their fate in detail, because they do not participate in the formation of the intestinal lining. In all echinoderms studied to date, the coelomic epithelium regenerates from its own cells [18,19,30,56,68–70]. The same is apparently true for *H*. *robustipinna*. In some cases, the coelomic epithelium can give rise to muscle cells or enterocytes [13,21,29], but we did not demonstrate participation of this epithelium in the formation of the gut luminal epithelium in *H*. *robustipinna*. It is worth mentioning that after artificially removing the visceral mass in the comatulid *A*. *mediterranea*, which is not capable of autotomy, enterocytes probably develop from coelomic epithelium cells [37]. In this case, JLCs do not participate in regeneration.

Amoebocytes containing large oval granules are the most conspicuous of the cells in the developing visceral mass. These cells do not participate in the formation of the lost organ; instead, they may help to provide conditions favouring regeneration. Amoebocytes migrate to the damaged area and are destroyed there, releasing granules. These granules are absorbed by all types of cells involved in regeneration. The contents of the granules may include nutrients and/or factors that cause activation of certain gene cascades [37,71].

The nature and the origin of JLCs still remain unknown. They were first described as neurosecretory cells in brittle stars [72]. However, the large size of their granules and evidence for high levels of calcium in these granules distinguish JLCs from typical neurosecretory cells [43]. Nevertheless, JLCs may form ganglion-like nodes and create synaptic contacts with nerve cells [43,62,73]. In addition, a neuropeptide, referred to as somatostatin, has been detected in them [48]. In this regard, they are now considered to be a special type of neurosecretory neuron [66] and, accordingly, may have an ectodermal origin. Thus, the transdifferentiation of ectodermal (nerve) cells into endodermal ones occurs during regeneration of the visceral mass in *H*. *robustipinna*. Transformation of nerve cells into other cell types is also known in other animals. In particular, mammalian neurons can transform into epithelial cells *in vitro* [74,75]. The reverse process, direct transdifferentiation of epithelial cells of the rectum into neurons, has been described in the nematode *Caenorhabditis elegans* (Maupas, 1900) [1,76]. In the examples above, transdifferentiation is performed within a single germ layer, the ectoderm. A more significant transformation of ectodermal cells is described for the budding tunicate, *Polyandrocarpa misakiensis* (Watanabe & Tokioka, 1972). The atrial epithelium of this species has an ectodermal origin; during budding it gives rise to all types of tissues of new zooids, including the digestive epithelium [12,77,78]. Unlike the cells of the atrial epithelium of ascidians, JLCs are not epithelial cells. To form the intestinal epithelium in *H*. *robustipinna*, they must additionally activate the epithelialization program, probably similar to that of mesenchymal-to-epithelial transition. A program like this is involved in neuronal differentiation during the development of the mammalian brain [79].

Animal cells can transform in two ways: direct transdifferentiation and transdifferentiation through a stage of dedifferentiation [1,75,80]. In *H*. *robustipinna*, the transformation of JLCs into enterocytes apparently represents an example of direct transdifferentiation. Destruction of type 2 granules should not be considered as a sign of dedifferentiation, as structure of the JLCs is not thereby simplified and the cells keep some features of their organization (type 1 granules).

#### The role of proliferation in regeneration of the gut in *H*. *robustipinna*

In case of regeneration of visceral mass in *H*. *robustipinna*, the proliferative activity is observed in different tissues. In the epidermis and coelomic epithelium of the gut, intensively EdU-labeled nuclei are found within 2–7 days post-evisceration. Mitoses in these epithelia are extremely rare. In the epidermis and coelomic epithelium, cells begin entering the mitotic cycle on day 2 post-autotomy and continue it during further regeneration. In the intestinal epithelium of the studied animals, DNA-synthesizing cells occur on day 2 post-autotomy and are not identified subsequently. The intensity of EdU labeling of nuclei is very low. Nevertheless, mitoses are observed in the luminal epithelium of the gut of these animals. According to Holland [81], the mitotic cycle during regeneration of the arms in *A*. *mediterranea* takes about 24 h, including an S period that lasts for 12.5 h. In this regard, it can be assumed that JLCs in *H*. *robustipinna* enter the mitotic cycle at earlier stages of regeneration, within 24–36 h post-autotomy, at the same time as their transformation. This assumption is confirmed by the data on the proliferative activity of cells during gut regeneration in *A*. *mediterranea* [37]. In this species, the first DNA-synthesizing cells are observed within 24 h after removal of the visceral mass.

However, keeping the animals in a colchicine solution for two days at different stages of organ formation did not arrest regeneration of the visceral mass. The exposure to colchicine during the first two days post-autotomy showed no effect on development of the intestinal lining, although proliferative activity was also observed in this period. Furthermore, we could not find mitotically dividing cells locked at the stage of prophase or anaphase, so-called c-mitosis, at any of the regeneration stages studied. This may be a consequence of the small number of mitoses in tissues of the forming visceral mass. Some slowdown of regeneration was manifested only as a lag in the differentiation of the enterocytes. The lack of effect of colchicine could be a result of the low dosage of this substance. However, the concentration we used (10^−7^ M) corresponds to that used in medicine to inhibit growth of tumors [82,83]. The retarded differentiation of the enterocytes could be attributed to the toxic effect of colchicine. Thus, our data obtained using EdU and colchicine indicate that cell division plays only a small role in the formation of the visceral mass.

The active migration and the minor contribution of proliferation to anlages being formed is probably one of the mechanisms that accelerate recovery. As is known, the number of cells at the site of injury grows faster due to migration rather than proliferation. Therefore, one of the first responses to injury (besides inflammation) in almost all animals is wound epithelialization by migrating cells [84,85]. For example, the mitotic activity of wound epithelium in vertebrates starts only on days 2–3 post-injury, when the wound is already epithelialized [86].

It should be mentioned that gut regeneration in *A*. *mediterranea* is accompanied by a noticeable proliferative activity [37]. As early as on day 2 after artificial removal of the visceral mass, mitotically dividing cells can be detected at the site of damage even by the conventional methods of electron microscopy. However, in spite of this high proliferative activity, the rate of regeneration in this species is lower than that of *H*. *robustipinna*. In *A*. *mediterranea*, development of the intestinal lining starts only on day 3 post-injury, and differentiation of enterocytes occurs within 5–7 days post-injury. These differences in the rate of regeneration are likely to be associated with different regeneration mechanisms in crinoids, which vary in the ratio between migration and proliferation, as well as in the origin and number of enterocyte precursor cells.

Thus, we have shown the unusual role of juxtaligament cells in crinoids. Their participation in regeneration and the capability of transdifferentiation allow us to take a new look at the origin and functions of these cells. Another feature of *H*. *robustipinna* is the fact that the blockage of proliferation does not affect regeneration. Similar data have recently been obtained for other echinoderms also [87,88]. These interesting features of regeneration in *H*. *robustipinna*, as well as the capability of visceral mass autotomy give grounds to consider this species as a convenient model object for studying mechanisms of morphogenesis and cell reprogramming.
